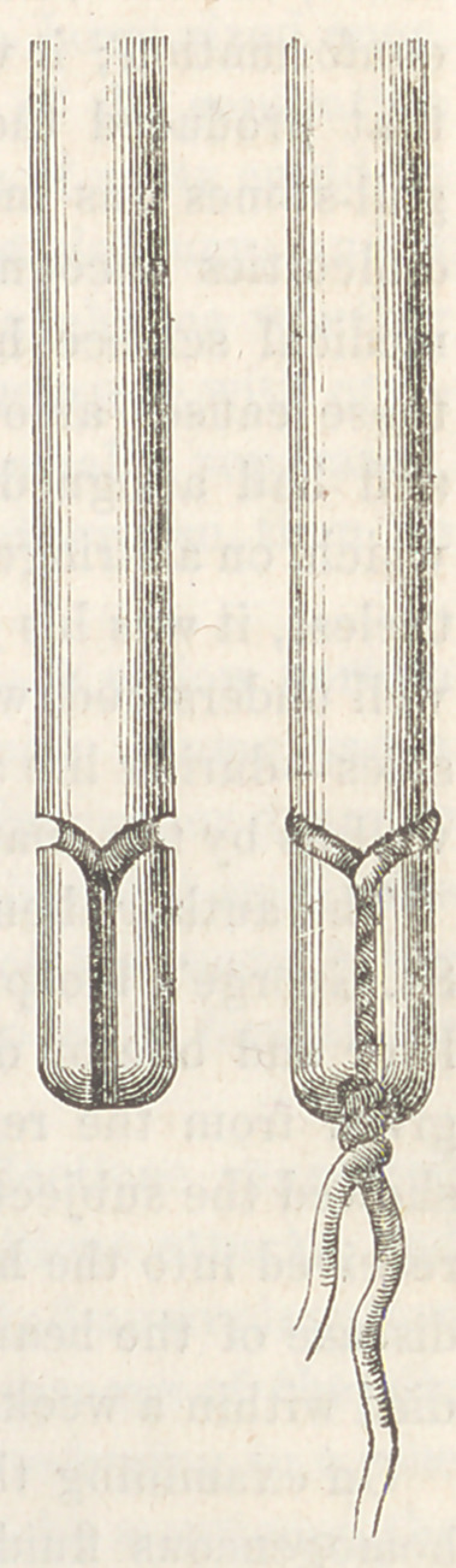# Surgery

**Published:** 1860-01

**Authors:** 


### SURGERY.

1. TRACHEOTOMY IN CROUP. By *Conway Evans, M.D.*—The author commenced by remarking upon the frequency and fatality of croup as a disease of early life, in illustration of which he observed that out of every thousand deaths of children between the ages of one and ten years, which occurred in England and Wales during the year 1856, sixty were due to this malady. He then proceeded to examine into the rate of mortality from croup, and pointed out the very slight measure of success which has hitherto attended the methods of treatment usually employed in this disease. The following cases—four of croup and two of diphtheria (?)—in which tracheotomy was performed, were then narrated in detail:—

*Case* 1.—A boy, aged nine; attacked with croup of slow accession; tempo rary amendment in the symptoms, followed by threatening suffocation; tracheotomy; death four hours after the operation; existence of crupous exudation down to the second and third subdivisions of the bronchi.

*Case* 2.—A girl, aged three; croup treated by leeches; counter-irritation, tartar emetic and calomel; tracheotomy on the fifth day, asphyxia being so complete as to render artificial respiration necessary; ejection of false membrane from trachea, and likewise two casts of small bronchial tubes; after-treatment of a freely supporting character; recovery perfect.

*Case* 3.—A boy, aged two; croup, between two or three days, treated with emetics; tracheotomy on the third day, suffocation being nearly complete; death during the operation; the crupous exudation found after death to extend down to the first subdivision of the bronchi.

*Case* 4.—A boy, aged two and a half; croup treated by tartar emetic; suffocation imminent on the fourth day from the accession of the croupous breathing; tracheotomy; death from exhaustion sixty-five hours after the operation; false membrane found after death to extend down to the fourth subdivisions of the bronchi.

*Case* 5.—A boy, aged five; diphtheria (?) coming on slowly and insidiously; breathing croupous on the seventh day; treated by emetics, counter-irritation, calomel, and compound antimonial powder; suffocation imminent on the eighth day; tracheotomy, followed by supporting treatment; ejection of a piece of false membrane; sudden accession of severe diarrhœa about thirty-six hours after the operation, and death from exhaustion. No post-mortem examination.

*Case* 6.—A boy, aged ten; diphtheria coming on very insidiously during nearly a month; treated by salines, and the application of a solution of nitrate of silver to the throat; supervention of croupous symptoms, treated by counter-irritation, leeches, antimony, calomel, and chlorate of potash; asphyxia impending; tracheotomy; and stimulating after-treatment; death, apparently from syncope, about twenty-six hours after the operation. After death a thick false membrane, separable from the subjacent mucous membrane only with considerable force, was found to line the larynx and trachea, and to extend to the bifurcation of the latter. It probably, indeed, passed down into the lungs; but an examination of these organs was not permitted.

Observing that, as in a large proportion of the fatal cases of croup, the disease destroys life by asphyxia, the author proceeds to inquire into the reasons why tracheotomy is so rarely resorted to for the relief of this malady in Great Britain, and traces this mainly to the influence of the strong opinions against the operation which have from time to time been pronounced by many great authorities, both British and American, whose views in reference to this point are cited. These opinions the author believes have no valid foundation, and are unworthy of the confidence generally placed in them; and to this conclusion he is led from four classes of considerations, which are examined in detail. These are—1. The high rate of mortality from croup, both with and without treatment. 2. The immediate cause of death in a large majority of the fatal cases of the disease, viz., asphyxia. 3. The recorded cases of croup in which tracheotomy has been resorted to in this country when the patient has been all but suffocated, and in which complete recovery has followed the operation. 4. The great success which has attended the performance of tracheotomy in croup in France, in which country it has been extensively practiced. If to each of these considerations its fair value be assigned, there can scarcely be any other conclusion but that it is incumbent upon the practitioner to give a fair trial to any method of treatment in croup which promises for its results a lower rate of mortality than obtains under the usual plans, and especially to tracheotomy.

The objects to be gained by the performance of tracheotomy in croup are next pointed out, stress being laid upon the fact that the operation affords time for the disease to run its course, (which would frequently not involve the destruction of life, except for the occurrence of asphyxia, which ought really to be regarded as a circumstance in the disease in a great measure accidental,) and for the administration of such remedies as may be deemed advisable. The physiological effects of the free admission of air into the lungs through an opening in the trachea in a child undergoing suffocation from croup, are then considered, and the cause of death in those cases in which life terminates by asphyxia is also examined; the practical conclusion arrived at being that, while the symptoms of suffocation may be relieved in almost all cases by the late performance of tracheotomy, they may be prevented in many by recourse being had to that proceeding early in the course of the malady.

The principal objections which have been urged against the performance of tracheotomy in croup are then considered in the following order. Tracheotomy in croup has been objected to: *a.* As unnecessary when there is spasmodic closure of the larynx, and as useless when false membrane exists in the windpipe without such spasmodic closure. *b.* As useless when the false membrane extends below the point at which the opening into the trachea would be made, and especially when the croupous exudation passes down into the bronchial tubes. *c.* As tending in itself to induce bronchitis and pneumonia—diseases which in themselves involve considerable risk to life. *d.* As having been actually attended with so little success as practically to render the operation unjustifiable. *e.* As very difficult of performance, and as involving in itself great danger to life. The real value of each of these objections is then carefully and fully examined in the order above given, the answer to the first objection being illustrated by the following case:—

*Case* 7.—A girl, aged three; croup treated by the warm bath and by tartar emetic, in spite of which the case progressed from bad to worse until the third day, when, while symptoms of asphyxia were being gradually developed and signs of exhaustion were becoming well marked, the patient suddenly fell back in bed and died with scarcely a struggle. After death, but before the post-mortem examination, tracheotomy was performed. A mass of false membrane was found, almost filling the larynx, and quite occluding the rima, and extending downward to the third ring of the trachea; but the lowest part of the croupous exudation was just above the top of the tracheotomy incision. No false membrane existed in any other part of the trachea. The results of tracheotomy for the removal of foreign bodies from the air-passages are then investigated, as well as those of the performance of this operation for the relief of other maladies than croup. But, as the statistical method of examining this subject is believed by the author to be productive of an impression upon the mind of the practical physician by no means so lasting as a narrative of the results of clinical observation, the following cases in which tracheotomy was performed for the relief of other diseases than croup are given in detail:—

*Case* 8.—A man, aged forty-three, came under observation when nearly exhausted from distressed breathing, dependent on syphilitic disease of larynx, (probably ulcerative;) tracheotomy, followed by a supporting plan of treatment; recovery; but, though able to return to his occupation, (a laborious one,) unable to breathe without the tube eight months after the operation.

*Case* 9.—A gentleman, aged seventy-two; nearly asphyxiated from spasmodic closure of the larynx, associated with some disease of that organ, (probably of a malignant character;) tracheotomy; recovery, as regards breathing; but, though living in a state of comparative comfort, unable to breathe without the tube nine months after the operation.

*Case* 10.—A man, aged thirty-six; suffocation impending from œdema of glottis; tracheotomy, followed by a highly supporting plan of treatment; recovery complete and rapid; voice also perfectly restored.

*Case* 11.—A girl, aged fifteen; nearly suffocated from œdema of the larynx supervening upon chronic disease of that organ, associated with "*lupus non exedens*" of face, lip, and thigh; tracheotomy; recovery, but inability to breathe on the withdrawal of the tracheal tube two months after the operation.

*Case* 12.—A lady, aged twenty-eight; œdema of glottis supervening upon tubercular disease of the larynx; suffocation imminent; tracheotomy; temporary recovery, the patient continuing to live in a state of comparative ease for five months after the operation, when death resulted from exhaustion consequent on the full development of the pulmonary phthisis.

*Case* 13.—A woman, aged twenty-three; œdema of larynx associated with syphilitic disease of that organ, treated by calomel and opium, in spite of which suffocation became imminent; tracheotomy, followed by supporting treatment; ejection of complete cast of bronchial ramifications of one lung; recovery complete, except as regards voice.

*Case* 14.—A girl, aged nineteen; sloughing of the soft palate and the back of the pharynx, of syphilitic origin; inability to swallow; supervention of œdema of glottis, and threatening suffocation; tracheotomy, followed by supporting treatment: the patient being fed for several weeks by the stomach-tube; recovery complete.

*Case* 15.—A boy, aged three and a half; foreign body in windpipe; tracheotomy; but no foreign body discovered; incisions in trachea enlarged, and windpipe fully examined on several occasions, but without success; eventually incisions made, not only through several rings of trachea, but also upward through both the cricoid and the thyroid cartilages, so that a finger could be readily passed from the trachea into the mouth, but still without the detection of any foreign body; ultimate recovery complete, and voice regained.

The conclusions deduced from all these considerations and facts is that tracheotomy, though frequently a difficult operation, is by no means so dangerous a proceeding as is commonly supposed. An inquiry is then instituted into the causes of the want of success which has attended the performance of tracheotomy in croup in this country, and this is attributed chiefly to the following circumstances, viz.:—

1. To the fact that tracheotomy has been very rarely indeed resorted to in croup in Great Britain, except as a last resort when other methods of treatment have been tried and found unavailing, and when the patient has become nearly asphyxiated. 2. To the fact that the treatment employed prior to the performance of the operation has almost always been of a more or less depressing kind, usually consisting in the exhibition of tartar emetic, ipecacuanha, calomel, the abstraction of blood, the use of the warm bath, etc. 3. To the fact that the after-treatment has generally not been of that supporting character which nature requires for the due upholding of the patient's strength until the phenomena of croup shall have had time to run their course; and to the difficulty experienced in commanding constant attention in the way of nursing and watching for some days after the performance of the operation. The author then strongly urges the propriety of having recourse to tracheotomy for the relief of croup early in the course of that disease, and immediately that the existence of false membrane in the windpipe can be satisfactorily determined, and emetics have been fairly tried; and for these reasons:—

*a.* Because tracheotomy tends to prevent the mode of death by which nearly all fatal cases of croup, in which this operation is not resorted to, terminate, viz., death by asphyxia. *b.* Because tracheotomy facilitates the ejection and removal of portions of false membrane from the windpipe. *c.* Because tracheotomy tends to prevent the exhaustion due to the extraordinary efforts of breathing almost always made by the patient in this malady. *d.* Because tracheotomy, by prolonging life, affords time both for the phenomena of the disease to run their course and for the administration of remedies and of means of support to an exhausted system. *e.* Because tracheotomy facilitates the employment of topical applications to the interior of the windpipe, upon which great reliance is placed by some practitioners. *f.* Because the early performance of tracheotomy in France has been attended with results which are admitted, even by the opponents of the operation, to have been far more favorable than when recourse has been had to this procedure as an ultimate expedient. The physiological and pathological differences between the condition of a child nearly asphyxiated by croup, and that of a man half strangled by some mechanical cause, are then pointed out; and the necessity which exists in the former case for the free employment of a supporting plan of treatment is clearly proved. The cause of death in those cases of croup in which a fatal termination ensues, notwithstanding the performance of tracheotomy, is next examined, and this is shown to depend upon one or more of the following conditions:—

1. On some accidental circumstance connected with the operation, such as hemorrhage into the windpipe, obstruction of the tube, etc. 2. On asphyxia dependent on the extension of the croupous exudation into the lungs, or on the reformation of the false membrane after its having been once ejected. 3. On complicated diseases (either connected with the operation or without any reference to it) arising in the course of the croup, such as bronchitis or pneumonia. 4. On exhaustion—death by asthenia.

The author believes that croup, when it proves fatal, always tends to destroy life by exhaustion, and that this would be its ordinary mode of termination were it not that the part of the body, in which the most striking alterations of structure induced by malady occur, is one in which the existence of such a mechanical obstruction as is presented by the croupous exudation tends, as well in itself, as in the spasmodic closure of the larynx, with which it is often associated, to destroy life by suffocation before the disease has had time, as it were, to run its full course and produce death by asthenia. And he, therefore, strongly advocates the propriety of adopting a supporting plan of treatment in this malady both before and after the operation, but especially after its performance. The value of alcohol, as a remedial agent in the treatment of disease, is then examined, and the method in which it should be given, viz., in small doses at short but regular intervals, is pointed out. Alcohol should be regarded, as has been remarked by Dr. Todd, not as a specific remedy, but simply as a kind of food. It is really a hydrocarbon, very easy of digestion, possessing certain properties of enabling the body temporarily to withstand exhausting influences, and capable, by its undergoing oxydation in the system, of maintaining the animal temperature and of preventing waste of tissue.

The *modus operandi* of the remedies usually employed in croup is then discussed, and their real value indicated; and the error of supposing this disease to consist in ordinary inflammation in the windpipe is alluded to. And, while the inefficiency of the remedies commonly used in croup is pointed out to be as theory would lead us to expect, the same fact is shown practically by the results of experience, which clearly indicate that under all plans of treatment, exclusive of tracheotomy, croup is a very fatal malady. The value of emetics is also examined, and the danger which frequently results from the employment of tartar emetic is dwelt upon. The circumstances which tend to diminish the chances of success from tracheotomy, are then referred to under the following heads:—

*a.* The age of the patient. *b.* The existence of pneumonia or bronchitis. *c.* The presence of other diseases, such as measles, hooping-cough, etc. *d.* The employment of depressing remedies prior to the operation. *e.* The postponement of tracheotomy until the patient is *in extremis*. *f.* The extension of the croupous exudation into the lungs.

After suggesting a few practical hints in connection with the operation itself, and in regard to the inhalation of chloroform in these cases; and, after briefly glancing at the various points which have been examined in detail, the author thus concludes: It only remains to warn the practitioner against expecting a large share of success from this operation, inasmuch as in our present inability to ascertain whether the croupous exudation is limited to a small portion of the windpipe, or whether it extends into the minute branches of the bronchial tree, we must necessarily oftentimes recommend its performance in cases in which death must almost inevitably take place. But, while a careful examination of this subject clearly indicates the propriety of making an opening into the trachea in those cases of croup in which false membrane exists, and of not postponing the operation until the last moment; and while it leads to the anticipation of a decided diminution in the rate of mortality from this disease when the early performance of tracheotomy is extensively practiced; the student of science cannot but feel that tracheotomy is at best but an expedient of relief, capable by its mechanical action of obviating certain tendencies to death, and by permitting the administration of support to an exhausted system, of affording time for the due occurrence of certain processes necessary to recovery. Nor can the practical physician forget that some effectual remedy for croup has still to be searched for, and not to be found, in all probability, until the true etiology and pathology of the disease are far better understood than at the present day. At the same time, it is impossible to foretell how near at hand the day may be when there shall be found a man who will do for croup what Jenner did for small-pox, or when there shall be discovered a remedy for this malady, as certain in its power and as efficacious in its action, as is iodide of potassium in syphilitic periostitis, or as is quinine in ague.—*Medical Times and Gazette*, August, 1859.

2. COMMENTS ON THE SERIES OF TRACHEOTOMY FOR LARYNGEAL AFFECTIONS (EXCLUSIVE OF TRUE CROUP.)—In this series we have 37 cases, 17 of which resulted in recovery, and 20 in death. If we classify the whole of the cases with regard to the nature of the disease for which the operation was required, we shall find that in 18 the patients had suffered from *syphilitic laryngitis*. Of these 18, 8 recovered, and 10 died. In 3 of the 8 cases of recovery, and possibly in one or two others, respecting which our notes do not extend over a sufficient length of time, the patient was never able afterwards to dispense with the canula. Of the 19 non-syphilitic cases; in 7, the disease was acute laryngitis; in 2, it was inflammation of the pharynx and tonsils, probably complicated with œdema of the larynx; in 1, it was laryngitis occurring in the course of typhoid fever; in 6, it was chronic laryngitis; in 2, it was inflammatory œdema, complicating phthisis; and in 1, it was suppuration beneath the mucous membrane of the glottis. We will briefly consider each of these groups separately.

*Acute Laryngitis.*—In this group we count 7 cases, of which 3 recovered, and 4 died. Of the fatal cases, in the first death occurred suddenly in a fit of delirium, ten hours after the operation. The autopsy showed the epiglottis in a state of gangrene. In the second, death occurred before the operation was completed. In this instance the symptoms of laryngitis had only been present a few hours. The autopsy showed œdema of the submucous tissue of the glottis. In the third death occurred on the fourth day; but we have no particulars as to its cause, or respecting the post mortem. In the fourth case, an old man, the subject of albuminuria, died during the performance of tracheotomy for the relief of acute laryngitis. The patients in the three cases which resulted in recovery were all able to dispense with the canula within ten days of the operation, and in all the wound subsequently healed well.

*Inflammation of Pharynx, Tonsils, etc.*—Of the two cases in which laryngotomy was necessitated by inflammation of the pharyngeal structures, one resulted in recovery and one in death. The fatal case was that of an old man, in whom death occurred on the third day. The autopsy showed purulent œdema about the pharynx and glottis, and purulent infiltration of the lungs. The case which recovered was that of a girl in whom the right tonsil had become so much swollen that it occluded the pharynx. Deep punctures had been made, but no matter had been obtained. Laryngotomy was performed, but the canula was not introduced. The wound healed in about a week. Three weeks from the commencement of the symptoms the abscess in the tonsil gave way, and "about half a pint of greenish well-formed pas" was brought up.

*Laryngitis occurring in Typhoid Fever.*—Our series presents only one instance which can be placed under this heading. The man died twenty-seven hours after the operation.

*Tracheotomy for Chronic Laryngitis.*—This group includes 6 cases. In one of these the operation was performed twice within an interval of a month. We may thus count them as seven operations, and from six of these the patients recovered perfectly. The case which ended fatally was that of a delicate woman, in whom the disease had existed for six weeks, and in whose case reducing treatment had been employed. She did well for the first four days, but sank under profuse diarrhœa on the fifth. Extensive disease of the thoracic viscera was found at the autopsy. Of the five cases which recovered, in two (if not in three) the patients were unable to dispense with the canula at the date of the last note. In one of them the laryngitis was probably connected with pulmonary mischief, as the patient subsequently died of phthisis, having, however, quite recovered as far as the operation was concerned.

*Tracheotomy for Laryngitis supervening during Phthisis.*—In this group we have two cases; in both the phthisis was advanced, in both the operation afforded great temporary relief, but both ended fatally within three days of its performance.

*Abscess beneath the Mucous Membrane of the Glottis.*—The subject of this case died on the third day after the performance of the operation. At the autopsy an abscess at the posterior part of the larynx was found which contained pus in which one of the arytenoid cartilages lay quite detached.—(*London Med. Times and Gazette,* October 29, 1859.)

3. Sulphuric Ether as an Anæsthetic; its first use in Massachusetts General Hospital.—Our readers are all familiar with the particulars of the introduction of anæsthetic agents into use, but the following pleasant account of the first use of ether in the Massachusetts General Hospital, will be read with interest. It is taken from an article by Prof. George Hayward, contributed to the last number of the British and Foreign Medico-Chir. Review:—

"It was my fortune to perform the first capital operation on a patient rendered insensible by the inhalation of sulphuric ether. This was done on November 7th, 1846, at the Massachusetts General Hospital, Boston. On September thirtieth preceding, Dr. Morton, a dentist, administered it to a man from whom he had extracted a tooth, without causing pain. Almost immediately after, he requested the late Dr. John C. Warren, who was at that time the acting surgeon at the hospital, to use it at that institution. Dr. Warren consented. It was inhaled by a patient, with partial success, on whom Dr. Warren operated on October sixteenth. The operation was the removal of a nævus from the face. On the day following I extirpated a large fatty tumor from the arm of a female, who was made wholly unconscious and insensible by the inhalation of the ether. The operation lasted seven minutes.

"At that time Dr. Morton was, I thought, the only person who knew what the anæsthetic agent was. On November first I took charge of the surgical department of the hospital, and in a day or two after Dr. Morton asked me if I were willing to allow him to administer his 'composition,' as he called it, to a female whose limb I was about to remove above the knee. I told him I would not, unless I knew what the article was, and felt confident of the entire safety of its administration. He at once told me that it was rectified sulphuric ether. He allowed me to communicate this to my colleagues, with an understanding that it should not be made known publicly, until he had obtained a patent, for which he had already applied. On the following day the operation was performed, in the presence of more than two hundred spectators.

"It rarely falls to the lot of a professional man to be the witness of a scene of more intense interest. The operating room was crowded. Many were obliged to stand. Besides the class of students in attendance on the lectures, numbering more than a hundred, and many of the principal physicians and surgeons of the city and neighborhood, there were present several clergymen, lawyers, and other individuals from the various callings of life. When I entered the theatre, before the patient was brought in, I found it, to my surprise, filled in every part, except the floor on which the table stood, with persons on whose countenances were depicted the almost painful anxiety with which they awaited the result of the experiment they were about to witness. I simply told them that I had decided, with the advice of my colleagues, to allow the patient, on whom I was to operate, to inhale an article which was said to have the power of annulling pain. The patient was then brought in. She was a delicate-looking girl, of about twenty years of age, who had suffered a long time from a scrofulous disease of the knee-joint. It had at length suppurated; there were extensive openings into the cavity of the joint; the cartilages were ulcerated and partly absorbed; the bones carious, and symptoms of hectic fever had already made their appearance. As soon as she was well arranged on the table, I told her that I should let her breathe something which, I hoped, would prevent her from suffering much from the operation, and that she need not be afraid of breathing it freely.

"As the ether was at the time administered by means of a large and clumsy instrument, which required to some extent the co-operation of the patient, it was desirable that the amputation should be done as rapidly as possible. Everything, therefore, was arranged with this view. I decided to perform the flap operation. One person was to compress the artery, another to withdraw the flaps, a third to hand the instruments, and a fourth to watch the pulse. I grasped the patient's limb with my left hand, and held the amputating knife behind me in my right, carefully concealed from her view. The mouth-piece of the inhaling instrument was then put into her mouth, and she was directed to take long inspirations. After breathing in this way a short time, the nostrils were compressed, so that all the air that went into the lungs must first pass through the machine, and of course be mixed with the vapor of the ether. She breathed with perfect ease, and without struggling, and in about three minutes from the time the instrument was put into her mouth, Dr. Morton said, 'She is ready.' A death-like silence reigned in the room; no one moved, or hardly breathed. I passed the knife directly through the limb, and brought it out as rapidly as I could, and made the upper flap. The patient gave no sign of feeling or consciousness, but looked like one in a deep, quiet sleep. Every other person in the room took a full inspiration that was distinctly audible, and seemed to feel that they could now breathe again. The second flap was then made, the bone sawed, five arteries were tied, and as I was tightening the ligature upon the sixth and last, she groaned, being the first indication of sensibility that had been given. Nothing more was done than to bring the flaps together, cover the stump with cloths dipped in cold water, and apply two or three turns of a roller to keep them in place. Her consciousness soon returned; she was wholly ignorant that the operation had been done. For some time she would not believe it, and said that she had felt nothing till I tied the last artery. The operation lasted a minute and three-quarters, not including the time required to tie the arteries. I did it rapidly, though it has been done in less time, because I feared that the insensibility might pass off, and we had no means then, as we have now, of continuing it as long as is necessary.

"Patients who have inhaled ether, when its effects are first passing off, are usually bewildered, not easily contented, and by no means inclined to do as they are desired. It would be almost impossible to persuade one of them at such a time to breathe through the instrument that was then in use. At present, fortunately, we can keep up the state of anæsthesia as long as we wish, by administering the agent employed for this purpose by means of a sponge. This simple contrivance was first used at the Massachusetts Hospital.

"The patient, whose case I have just spoken of, recovered rapidly from the operation, was in good health when I left home eleven years after, and I have no reason to suppose that she is not so at the present time.

"It will be readily believed that a result so successful, and witnessed by so many intelligent persons, made it impossible to doubt the anæsthetic power of the agent employed, and what this was very soon became known. In an almost incredibly short space of time, numerous operations were performed on persons rendered insensible by the inhalation of ether, in various parts of the United States and Europe, and there is hardly a country in Christendom in which it has not been thus used to a greater or less extent."—(*Cincinnati Lancet and Observer*, December, 1859.)

4. EXTRACTION OF CATARACT BY LINEAR INCISION. (From the Quarterly Report of Cases occurring at the Edinburgh Eye Infirmary. By *Benj. Bell, Esq., F.R.C.S.E.*, and *Patrick Heron Watson, Esq., M.D., F.R.C.S.E.*)—During the last three months, two soft cataracts have been removed by what has been called the operation of linear incision. The word linear is probably used in contradistinction to the flap, which is made by the incision in the ordinary operation of extraction, where it involves nearly one-half of the cornea. The proceeding of which we are about to speak might be more properly named extraction through a small section.

The chief advantages of the operation seem to be these two: 1. It affords an easy and expeditious mode of getting rid of soft cataracts, to which alone it is applicable, for it is well known that when these are treated in the ordinary way, by being broken up with a view to solution, they are often dissolved very slowly and not without ultimate injury to the visual powers of the eye. Ophthalmic surgeons are familiar with the observation, that the process of solution is not unfrequently followed by a marked impairment of vision, as if the deeper and more important textures had, somehow or other, been interfered with in their nutrition, by the prolonged and exhausting effort to dissolve and remove the opake lens. But, by removing the cataract at once, as in the operation by *linear incision*, the organ is spared this effort at solution, and the likelihood of vision being restored is greatly increased. And even if a portion merely of the cataract escapes through the opening in the cornea, the remainder dissolves and disappears more rapidly and with less injury to the eye than if the whole lens had been allowed to remain in the chambers of the aqueous humor, after being broken up by the needle. 2. The smallness of the incision of the cornea—which, however, the cataracts being soft, is amply sufficient—renders the operation both less dangerous and more easily performed than the ordinary method of extraction for cataracts of hard and firm textures. Such cataracts always require an ample section of the cornea; and, under ordinary circumstances, and in suitable cases, no other operative procedure, notwithstanding the difficulty of its performance, can bear a comparison with it.

The operation by linear incision may be performed in the following manner: But, first of all, is chloroform to be administered? There seems to be less objection to its use in this operation than in ordinary extraction with a large division of the cornea; because, in the event of sickness and vomiting being induced, the risk of injury to the eye is obviously smaller. Moreover, unless the self-possession and steadiness of the patient can be relied on, there will be considerable advantage from inducing anæsthesia, if the scoop should require to be frequently employed in removing broken portions of the lens from the anterior chamber. Preliminaries being over, the incision of the cornea is made by means of a triangular-shaped knife, sharp at the point, keen on both edges, and about two and a half or three lines broad at its base. It should enter the cornea near its outer margin, and pass horizontally in front of the iris, until the whole length of the cutting edges has penetrated the anterior chamber. The knife is then withdrawn, care being taken that as little as possible of the aqueous humor be allowed to escape. The next stage of the operation consists in passing a fine cutting needle into the opening, freely dividing the capsule, breaking up the substance of the lens, and bringing the fragments into the anterior chamber. Most of the latter will, in all probability, be carried out by the aqueous humor, as it gushes through the wound of the cornea; but, if this should not be the case, we withdraw the needle, and with a small silver scoop, made for the purpose, endeavor carefully and gently to effect the same object. But, as we have already hinted, there is no serious objection to our allowing small fragments of the cataract, which is supposed to be of soft texture, to remain in the anterior chamber; for the wound in the cornea speedily heals, the aqueous humor is resecreted, and the remaining portions will rapidly disappear. It is better far to trust to this course of events, than to give way to the *nimia diligentia chirurgorum*, which, by bruising the edges of the wound, might prevent it from healing, and perhaps occasion serious inflammation of the iris with all its consequences.

Some prefer breaking up the cataract, in the first place, with a very fine cutting needle, so accurately made as not to let out the aqueous humor, and then making the incision of the cornea in the manner already described. The objections to this are of a practical nature: the extreme difficulty of withdrawing the needle without allowing a little of the aqueous humor to escape, and then the ulterior difficulty, of cutting in a satisfactory manner with the triangular knife the imperfectly distended cornea. To obviate this latter difficulty, the second part of the operation might be postponed until the following day, when the aqueous humor would be resecreted; but then there might be some risk of inflammatory action being kindled by the portions of lens which had been brought into the anterior chamber. No general rule need be laid down. The principle of the operation being kept in view, the details may be modified in individual cases, according to the judgment of the surgeon.—(*Edinburgh Medical Journal*, September, 1859.)

5. NEEDLE FOR THE WIRE SUTURE. By *R. J. Levis, M.D.*, Surgeon to the Philadelphia Hospital.—The present general use of the metallic suture in surgery will make acceptable a form of needle adapted to its peculiarities, and which, by facilitating its introduction through the tissues, will add to its convenience and efficiency.

For the free passage of the needle and wire, it is essential, first, that the wire be securely held; second, that it present, at its connection with the needle, no impediment to the transit through the tissues; third, that it should follow the needle in a direct line, not allowing an angle to form at the junction which will require traction to overcome. It is also important, for the convenience of the operator, that the needle should be readily threaded.

In using the ordinary surgical needle for the purpose, even though the needle be deeply grooved at the eye for its lodgment, the wire forms a ring-like attachment with the needle, which impedes its passage. A sort of hinge joint is also formed at the junction, which will be movable, no matter how tightly the wire be twisted, and there will be continually forming an angle with the needle, which is a great impediment to its use in delicate tissues, and in regions difficult of access, as the vagina, rectum, and fauces. Another inconvenience which has occurred with me in the use of the ordinary needle and the silver wire, is the liability of the wire to be pinched off by the forceps necessary to its introduction in the above localities.

These objections have induced some surgeons to introduce the wire by first passing the ordinary silk thread to which the wire is attached, and then drawn through.

For the purpose of overcoming these inconveniences in the ordinary use of the wire suture, I have devised a modification of the needle, which is so well illustrated by the accompanying proportionally colossal representations, as to render description almost unnecessary.

Its peculiarity is in what is usually the eye of a needle, though this is really without an eye, the attachment of the wire being accomplished by grooves, in which it rests.

A groove, deep enough for the lodgment of the wire, encircles the needle obliquely near its extremity, and leads into another groove, which is vertical. The vertical groove is just wide enough at its entrance to admit the introduction of one wire at a time; but the inside of the groove being large enough to accommodate two wires, when both are introduced and twisted together they are securely held. An attachment is thus effected which is as firm as if the needle and wire formed one continuous piece, and the wire being entirely incased within the grooves it will traverse any tissue of the body without the least impediment.

There is a decided advantage in having the wire double for an inch or more following the needle, as any break in the wire invariably occurs very near to the needle.

This form of needle has been so extensively used in this city as to thoroughly test its efficiency, and it has been preferred by the instrument makers on account of its simplicity and the facility and cheapness with which it can be made.

Such needles, of sizes adapted to all uses, may be had from Mr. Gemrig, of Eighth Street, or Mr. Kolbé, of Ninth Street, in this city.—(*Medical and Surgical Reporter*, December 3, 1859, p. 224.)

## Figures and Tables

**Figure f1:**